# Power of selective genotyping in genome-wide association studies of quantitative traits

**DOI:** 10.1186/1753-6561-3-s7-s23

**Published:** 2009-12-15

**Authors:** Chao Xing, Guan Xing

**Affiliations:** 1 Department of Clinical Sciences, University of Texas Southwestern Medical Center, Dallas, Texas 75390, USA; 2 McDermott Center of Human Growth and Development, University of Texas Southwestern Medical Center, Dallas, Texas 75390, USA; 3 Bristol-Myers Squibb Company, 311 Pennington-Rocky Hill Road, Pennington, New Jersey 08534, USA

## Abstract

The selective genotyping approach in quantitative genetics means genotyping only individuals with extreme phenotypes. This approach is considered an efficient way to perform gene mapping, and can be applied in both linkage and association studies. Selective genotyping in association mapping of quantitative trait loci was proposed to increase the power of detecting rare alleles of large effect. However, using this approach, only common variants have been detected. Studies on selective genotyping have been limited to single-locus scenarios. In this study we aim to investigate the power of selective genotyping in a genome-wide association study scenario, and we specifically study the impact of minor allele frequency of variants on the power of this approach. We use the Genetic Analysis Workshop 16 rheumatoid arthritis whole-genome data from the North American Rheumatoid Arthritis Consortium. Two quantitative traits, anti-cyclic citrullinated peptide and rheumatoid factor immunoglobulin M, and one binary trait, rheumatoid arthritis affection status, are used in the analysis. The power of selective genotyping is explored as a function of three parameters: sampling proportion, minor allele frequency of single-nucleotide polymorphism, and test level. The results show that the selective genotyping approach is more efficient in detecting common variants than detecting rare variants, and it is efficient only when the level of declaring significance is not stringent. In summary, the selective genotyping approach is most suitable for detecting common variants in candidate gene-based studies.

## Background

The extreme sampling strategy in quantitative genetics is termed selective genotyping [1], involving phenotyping a large population of individuals, but genotyping only those individuals whose phenotypes deviate substantially from the mean. This approach was first introduced in a linkage context and was readily adopted in association analysis by treating the upper and lower subpopulations as case and control groups, respectively.

This selective genotyping approach was shown to be efficient in association mapping of quantitative trait loci (QTLs) in a statistical framework [2,3]. Since then, there have been several theoretical studies proposing alternative test statistics [4,5] or alternative sampling procedures [6], and extending this approach to haplotype analysis [7]. It is important to note that the selective genotyping approach was initially proposed to increase the power of detecting rare alleles with large effect at QTLs, but all the theoretical work cited assumed an unrealistic assumption of a large sample size even in the subpopulation of each extreme. The selective genotyping design has been applied to identify association between quantitative traits and genetic markers. For example, it was applied in studying genetic association of intelligence scores [8], attention-deficit hyperactivity disorder [9], and body mass index [10]. It is worth noting that in all of the studies mentioned previously that had positive results, the alleles of interest were common (minor allele frequency, or MAF > 0.20).

Both theoretical and applied studies to date have focused on limited number of loci in a candidate gene study scenario, and therefore their test levels were set high. They made no comparison between common and rare variants in a realistic situation in terms of sample size. In this study we investigated the empirical power of selective genotyping using the Genetic Analysis Workshop 16 (GAW16) rheumatoid arthritis (RA) genome-wide association study (GWAS) data from the North American Rheumatoid Arthritis Consortium (NARAC). The genome-wide data provided us an opportunity to investigate the power at different test levels and a full spectrum of allele frequency.

## Methods

The NARAC data consists of 868 cases and 1,194 controls. The samples were genotyped using the Illumina Infinium HumanHap550 chip, and in the current study we focused on 531,689 single-nucleotide polymorphisms (SNPs) across the autosomal genome. The data were filtered through genotype call rate (>90% completeness) and the Hardy-Weinberg equilibrium test (*p*-value > 0.001), and 522,587 SNPs were retained. Note that we did not filter the data through a MAF criterion because the MAF was a primary variable we were investigating. To control for population stratification, we calculated the first two principal components, denoted PC1 and PC2, of 1,344 ancestry-informative SNPs [11] and adjusted for them together with sex in the subsequent association analysis. Because all individuals were genotyped, we chose a proportion of the whole sample to mimic the selective genotyping procedure. Given a quantitative trait, the upper *N*^th^, *N *∈ {10, 20, 30, 40, 50, 60, 70, 80, 90, 100}, percentiles of cases, and, correspondingly, a random *N *percent of controls were selected as a study dataset, i.e., there were 10 datasets generated in total. All subjects were included when *N *= 100. A total of three RA-related traits, including a binary variable of RA affection status and two quantitative phenotypes (anti-cyclic citrullinated peptide, or anti-CCP and immunoglobulin M, or IgM) measured only in cases were used in the analyses.

The statistical testing procedure included three steps. First, we tested association between affection status and genotype using both cases and controls by a logistic regression model, which yielded a statistic *T*_1_. Second, we tested association between a quantitative trait and genotype in the selected cases by a linear regression model, which yielded a statistic *T*_2_. Because the distribution of a quantitative trait in the selected cases might not be normally distributed, the Box-Cox power transformation [12] was always performed in the linear regression. Covariates PC1, PC2, and sex were adjusted in both tests. Third, because *T*_1 _and *T*_2 _were independent under the null hypothesis of no association between the traits and genotype, we employed the Fisher's method of combining *p*-values [13] to form a statistic , where *P*(*T*_*i*_) denotes the *p*-value corresponding to the test statistic *T*_*i*_. We concentrated on results based on *T*_3 _in the subsequent analysis because it was shown to be more powerful than the other two [2].

To investigate the empirical power of selective genotyping in GWAS, we only focused on SNPs that attained a *p*-value < 0.05 under control of the false-discovery rate (FDR) [14] when using the whole sample. Setting the results using the whole sample as the gold standard, we defined relative power as, at a certain test level α, the total number of significant SNPs using a subset of data (*N *< 100) divided by the total number of significant SNPs using the whole data (*N *= 100). We studied the relative power as a function of three sets of parameters: the sampling proportion *N*%, *N *∈ {10, 20, 30, 40, 50, 60, 70, 80, 90, 100}, the test level α = 5.0 × 10^0^, *O *∈ {-2, -3, -4, -5, -6, -7, -8}, and the SNP MAF stratified by *p*, *p *∈ {0.05, 0.10, 0.20, 0.30, 0.40}. All analyses were performed using the software PLINK [15] and R [16].

## Results

We first performed a genome scan using the whole sample, and were able to detect loci confirmed to be associated with RA such as the major histocompatibility complex on chromosome 6, *PTPN22 *on chromosome 1, and *TRAF1-C5 *on chromosome 9 (data not shown), which proved the validity of the current method. To attain a *p*-value < 0.05 under FDR control, the nominal *p*-values for anti-CCP and IgM were 2.35 × 10^-6 ^and 2.34 × 10^-6^, respectively. There were 336 SNPs for anti-CCP and 337 SNPs for IgM that met these criteria. Their distributions across chromosomes and allele frequency categories are summarized in Tables 1 and 2. There were no SNPs with a MAF < 0.01 that met the criteria, and therefore the lowest threshold of MAF to stratify SNPs was 0.05.

**Table 1:** Genomic distribution of SNPs attaining a *p*-value < 0.05 under control of the false discovery rate using the whole sample

Chromosome	anti-CCP	IgM
1	2	3
2	1	0
3	3	1
5	3	2
6	313	318
9	7	7
12	1	0
14	0	1
15	1	0
16	3	3
17	2	1
18	0	1

Total	336	337

**Table 2:** Allele frequency distribution of SNPs attaining a *p*-value < 0.05 under control of the false discovery rate using the whole sample

MAF	anti-CCP	IgM
0.40 ~ 0.50	67	70
0.30 ~ 0.40	79	78
0.20 ~ 0.30	51	52
0.10 ~ 0.20	101	100
0.05 ~ 0.10	35	33
< 0.05	3	4

Total	336	337

**Figure 1:**
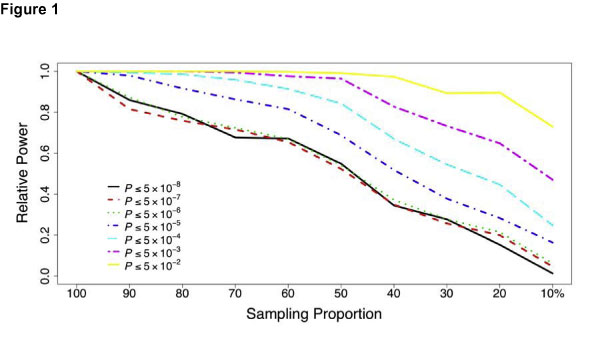
**Relative power of selective genotyping at different test levels for trait anti-CCP**.

**Figure 2:**
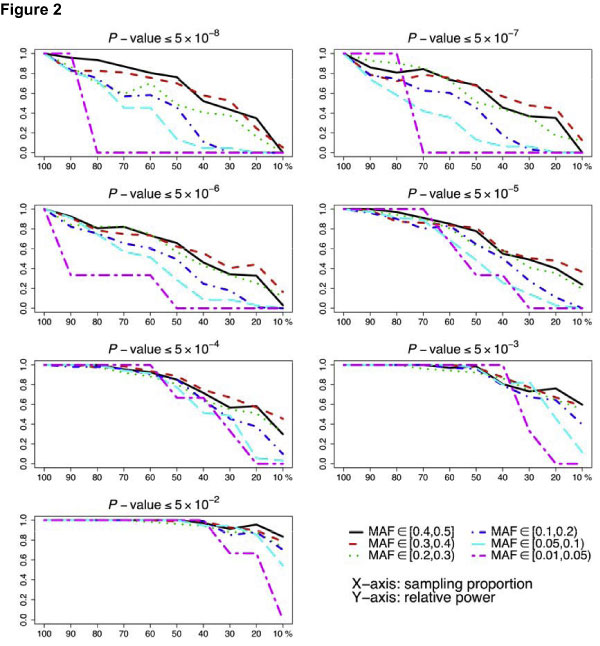
**Relative power of selective genotyping at different test levels stratified by the minor allele frequency for trait anti-CCP**.

The power of selective genotyping strategy depended on the test levels. This approach was efficient only when the level of declaring significance was not stringent. To achieve a relative power greater than 0.8, less than 30% of the sample needed to be genotyped when the test level was equal to or greater than 5 × 10^-3^; however, more than 50% of the sample needed to be genotyped at the test level of 5 × 10^-5^; when the test level was equal to or less than 5 × 10^-6^, the power decreased linearly with the sample size (Figure 1). Stratifying each line in Figure 1 by the MAF, we found the efficiency of selective genotyping increased as the MAF increased regardless of test levels (Figure 2). In other words, this approach was most efficient in detecting common variants. We observed similar results for both traits, and only presented those for anti-CCP.

## Discussion

Selecting individuals with extreme phenotypes increases the narrow sense of heritability of the trait in the sample at the cost of decreasing sample size. Therefore, there is a trade-off between these two factors to achieve a certain level of statistical power. This approach is more efficient in detecting common variants than detecting rare variants because the effective sample size (the number of variants in a sample) decreases more rapidly for rare variants as we decrease the sampling proportion.

Selective genotyping is commonly practiced by genotyping individuals with extreme phenotypes at both upper and lower extremes; however, in the current study we only selected those at the upper extremes in cases and then made random selection in controls. This study design was based on the trait characteristics, not constraints of the data available. Both anti-CCP and IgM are usually present only in cases, i.e., both quantitative traits are zero in unaffected persons. Therefore, the study population approximates a selected sample plus a random sample from the general population, similar to the design proposed by Slatkin [2].

## Conclusion

The selective genotyping approach is more efficient in detecting common variants than detecting rare variants even though it was initially proposed to detect the latter [2]. This approach is efficient only when the level of declaring significance is not stringent; therefore, it should not be employed in a GWAS, though it can be used in a replication study to confirm findings of the GWAS. In summary, the selective genotyping approach is most suitable for detecting common variants in candidate gene-based studies.

## List of abbreviations used

anti-CCP: Anti-cyclic citrullinated peptide (anti-CCP); FDR: False-discovery rate; GAW16: Genetic Analysis Workshop 16; GWAS: Genome-wide association study; IgM: Immunoglobulin M; MAF: Minor allele frequency; NARAC: North American Rheumatoid Arthritis Consortium; QTLs: Quantitative trait loci; RA: Rheumatoid arthritis; SNP: Single-nucleotide polymorphism.

## Competing interests

The authors declare that they have no competing interests.

## Authors' contributions

CX conceived of the study, participated in data analysis, and drafted the manuscript. GX participated in study design, carried out data analysis, and helped to draft the manuscript. Both authors read and approved the final manuscript.
